# m^6^A RNA methylation in brain injury and neurodegenerative disease

**DOI:** 10.3389/fneur.2022.995747

**Published:** 2022-09-08

**Authors:** Jianhui Deng, Xiaohui Chen, Andi Chen, Xiaochun Zheng

**Affiliations:** ^1^Department of Anesthesiology, Fujian Provincial Hospital, Shengli Clinical Medical College of Fujian Medical University, Fuzhou, China; ^2^Fujian Provincial Key Laboratory of Emergency Medicine, Fujian Provincial Key Laboratory of Critical Care Medicine, Fujian Provincial Co-Constructed Laboratory of “Belt and Road,” Fujian Emergency Medical Center, Fuzhou, China

**Keywords:** N6-methyladenosine, post-transcriptional RNA modification, RNA, brain injury, neurodegenerative disease

## Abstract

N^6^-methyladenosine (m^6^A), the most prevalent post-transcriptional RNA modification throughout the eukaryotic transcriptome, participates in diverse biophysiological processes including cell fates, embryonic development and stress responses. Accumulating evidence suggests that m^6^A modification in neural development and differentiation are highly regulated processes. As RNA m^6^A is crucial to protein translation and various bioprocesses, its modification dysregulation may also be associated with brain injury. This review highlights the biological significance of m^6^A modification in neurodegenerative disease and brain injury, including cerebrovascular disorders, is highlighted. Emphasis is placed on recent findings that elucidate the relevant molecular functional mechanism of m^6^A modification after brain injury and neurodegenerative disease. Finally, a neurobiological basis for further investigation of potential treatments is described.

## Introduction

**Figure 1:**
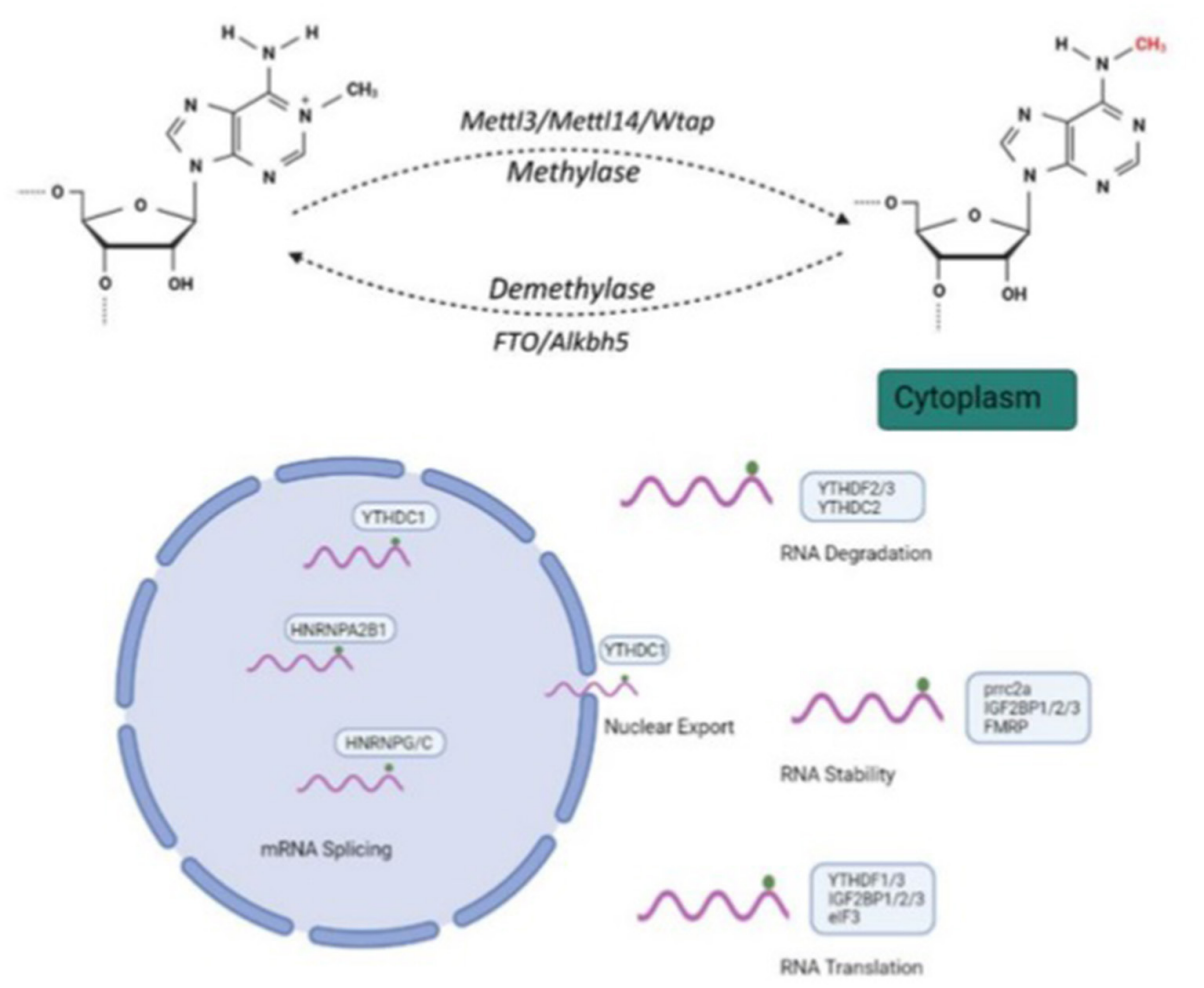
Molecular mechanism and three N6-methyladenosine(m6A) modification factors. m6A modification regulates RNA nuclear exports, stability, degradation, splicing and translation. Methyltransferase complexes, including METTL3, METTL14 and WTAP, as well as m6A RNA demethylases, FTO and ALKBH5, participate in m6A modification processes. Created with BioRender.com.

N^6^-methyladenosine (m^6^A) modification, the most common RNA post-transcriptional modification, plays an important role in gene expression by regulating processes like RNA nuclear export, stability, degradation, splicing and translation (Figure 1). This dynamic nucleus-based RNA process occurs primarily on adenine in the RRACH sequence (R = G or A; H = A, C or U) (1, 2).

The distribution and motif of m^6^A modification vary across tissue type and are expressed in a spatiotemporal-specific pattern regulated by methyltransferase complex and demethylases in various neurodevelopment and pathological processes (3–5). m^6^A modification is widely enriched in specific tissues including the brain, liver and kidney; It is less abundant in the fetal brain but developmentally increases in brain tissue (6).

m^6^A modification plays a pivotal role in the diversification of embryonic and adult neurons, and in the regulation of neural progenitor capacity (5). m^6^A modification is essential for nervous system functioning and development (4, 7–9). Altered m^6^A modification levels in the brain are closely related to learning, long-term memory and brain-injuring diseases (10–12). This systematic review focuses on the research to date into the mechanism of epitranscriptional m^6^A modification in neurological diseases.

## Methods for detecting m^6^A RNA methylation

In 1974, m^6^A was detected in poly(A) RNA fractions; however, the lack of epitranscriptomic mapping methods for m^6^A reduced researcher interest. With the development of several methods incorporating next-generation sequencing techniques, regulation and functions of m^6^A modification have recently been revealed. The early mapping approach (termed “Me-RIP-seq”) provided the first comprehensive analysis of methylated mRNAs throughout the transcriptome (13), including application of methylated RNA immunoprecipitation and chromatin immunoprecipitation-sequencing, showing that m^6^A is abundant in coding sequences in long exons, particularly in the vicinity of the stop codon (14). Using mi-CLIP, individual m^6^A sites were detected in cellular mRNAs with high sensitivity and specificity (15). Similarly, mi-CLIP enabled differentiation between m^6^A and m^6^Am, which forms a 2-O-methylated residue adjacent to the 7-methylguanosine cap structure (16, 17). Yet there are constraints on existing methods, including high input RNA requirements and cross-reactivity to other RNA modifications. Thus, several advanced methods, including deamination adjacent to RNA modification targets (DART-seq), m^6^A-label-seq and m^6^A-SEAL, which detect mRNA m^6^A transcriptome-wide at base resolution through metabolic labeling, have been proposed (18–20). Development of these methods may allow a better understanding of the mechanism of m^6^A modification.

## Highly specific brain tissue m^6^A methylome

Compared with non-brain tissues, brain tissues show highly specific m^6^A methylome, suggesting its involvement in regulation of brain-specific functions. m^6^A modification shows ubiquitous presence in the cerebral cortex and remarkable increases during neural development. AlkB homolog 5 (ALKBH5), the main demethylase, exhibits tissue-specific regulation in the brain and is highly enrich in the cerebellum and olfactory bulb (7). Likewise, expression of fat mass and obesity associated protein (FTO), another demethylase of RNA, remains high in neurons and exerts its regulating effect on neurophysiological processes, including neuronal differentiation, learning and memory and neurogenesis (21, 22). m^6^A modification of brain tissue RNA stop codon in the forms of “writer,” “eraser,” and “reader” of are widely expressed in the cerebellum and cerebral cortex, where they are required for the control of delicate gene expressions and thus form tissue-specific regulators that play indispensable roles in nervous system activities (22).

## Writers, readers and erasers of m^6^A modification

m^6^A modification is dynamically regulated by a multicomponent methyltransferase complex, including methyltransferase-like 3 (METTL3), methyltransferase-like 14 (METTL14) and Wilms tumor 1-associated protein (WTAP), as well as the recently identified m^6^A RNA demethylases, FTO and ALKBH5 (1). METTL3 exhibits catalytic activity of the m^6^A methyltransferase, in which it transfers a methyl group from S-adenosylmethionine (SAM) to an adenosine in RNA (23). ALKBH5 is regarded as a major eraser of m^6^A modification, which has been found of m^6^A demethylase activity. The YTH family comprises numerous proteins containing the YTH domain, which selectively bind the m^6^A in RNA, and m^6^A exerts its effects by recruiting m^6^A-binding proteins (24). RNA methylation levels are finely balanced by an interplay between m^6^A methyltransferases (writers), demethylases (erasers) and binding proteins (readers).

## m^6^A methyltransferase complex

### METTL3

It is reasonable to speculate that METTL3 is involved in mammalian brain development, based on immunostaining of various brain regions in which METTL3 is abundant. Conditional knockout of METTL3 results in smaller body size, ataxia-like movement disorders and early death in mice. In addition, abnormal upregulation of pivotal developmental genes, like Atoh1 and Cxcr4, and apoptosis-associated genes, like Dapk1 and FADD, account for regulation of mRNA stability induced by m^6^A depletion in CKO mice, which significant impacts survival and apoptosis of cerebellar cells. Further, Grin1, a synapse-related gene, is abnormally spliced in CKO mice, causing excessive calcium influx and ultimately apoptosis. This cumulative evidence confirms that METTL3 plays a critical role in cerebellar neuronal connection, survival and maturation (25). Furthermore, as it shown in the METTL3-knocked down cerebellum, apparent morphology changes are also observed in overexpression of METTL3 (5).

### METTL14

A heterodimeric enzyme complex composed of METTL3 and METTL14 lead to m^6^A modification of RNAs. In particular, the specific function of METTL14 is to support catalytic activity of methyltransferase of METTL3 in substrate recognition (23). Furthermore, the crucial role of METTL14 in the adult mammalian nervous system has been further confirmed by the significance of m^6^A in maintenance of normal nervous system functioning. As neural excitability is elevated by METTL14 deletion in the mouse striatum, striatal-mediated behaviors are significantly impaired, impacting learning and initial acquisition of response and sensorimotor learning (26). METTL14 is crucial for the regulation of gene expression during nervous system development; its deletion in neural progenitor cells and neural stem cells (NSC) can result in disruption of cortical development, in the forms of decreased proliferation and premature differentiation, respectively (27–29). Furthermore, embryonic mouse brains with knockdown METTL14 exhibit an extension of the cell cycle of radial glia cells and a period of cortical neurogenesis (27). Other studies have shown impaired injury-induced protein translation and functional axon regeneration in dorsal root ganglion (DRG), and CNS hypomyelination, from METTL14 knockdown (28–30). These studies suggest that METTL14 modulates diverse physiological and pathophysiological brain functions, including learning, cell proliferation and differentiation and CNS hypomyelination, providing new insights into the molecular mechanisms and treatments of brain diseases.

### WTAP

WTAP modulates gene expression and alternative splicing without catalytic activity through its interaction with the METTL3–METTL14 complex, which facilitates recruitment of the m^6^A methyltransferase to RNA targets, containing the consensus m^6^A motif, which affects cellular m^6^A deposition and localization into nuclear speckles (31). WTAP is required for normal differentiation processes, so that WTAP knockdown zebrafish embryos exhibit differentiation defects and increased apoptosis (32). Thus, there is strong evidence that WTAP may play a role as a regulatory subunit of the m^6^A methyltransferase complex, providing insight toward further investigating the fundamental mechanisms of the regulation functions of m^6^A.

### m^6^A demethylases

#### ALKBH5

Recent studies have advanced our understanding of the indispensable role of ALKBH5 in cerebellar development (5, 7). Brain and cerebellum sizes of ALKBH5-deficient mice are reduced, and in immunostaining analyses the numbers of Ki67+proliferating cells and phosphohistone3 (PH3+) mitotic cells in the EGL increased markedly compared with WT counterparts. Expanding cell numbers in the S phase of the cell cycle, reflected by immunostaining analysis, and mitotic cells have also been observed, denoting untimely and aberrant methylation induced by ALKBH5 deficiency, which hinders normal cerebellar cell proliferation and differentiation (5).

#### FTO

FTO is highly enriched in neurons and expressed in NSCs, which are also found most abundantly in the brain (33). Recently, FTO was revealed to be a regulator of proliferative and differentiated processes, but not apoptosis, in adult NSCs *via* modulating the Pdgfra/Socs5-Stat3 pathway, the products of which are involved in modulation of the stat3 pathway that participates in regulation of neurogenesis, regulation of which the BNDF pathway also participates (21, 34). Thus, FTO deficiency may lead to learning and memory impairments (21). A case report showed that homozygous mutations in FTO exhibits growth retardation and developmental delay, further confirming that FTO mutations may lead to early-onset neurodevelopmental disorders and suggesting that FTO regulates the activities of genes involved in key central nervous system development processes (35). After transient forebrain ischemia, FTO expression in the CA1 and CA2 is dramatically reduced, possibly contributing to hippocampal dysfunction (36).

### m^6^A-binding proteins: Focus on YTH domain protein and Prrc2a

#### YTH domain protein

Several recent studies have revealed that m^6^A RNA metabolism regulation effects, including mRNA splicing, RNA folding, translation, decay, stability and nuclear export, primarily depend on the function of YT521-B homology (YTH) domain-containing proteins which could be recruited to mRNA and directly recognize the m^6^A site through well-characterized YTH domains (37). Collectively, such YTH domain-containing proteins are: YTH domain-containing family protein (YTHDF), YTH domain-containing protein 1 (YTHDC1) and YTH domain-containing protein 2 (YTHDC2), which are evolutionarily conserved and involved in functional regulations such as translation efficiency and stability of m^6^A-containing RNAs (38–40). Moreover, YTH domain-containing proteins are required for pre-crossing axon guidance in spinal cord and neuronal survival improvement after ischemia brain injury and during neural development, raising the possibility that YTH proteins may play a critical role in brain structural integrity and physiological function (41–43).

#### Prrc2a

Advanced research has confirmed Prrc2a as a novel m^6^A reader that controls OPC specification, differentiation and myelination by regulating expression of olig2, which has a oligodendroglial lineage determination function, in an m^6^A-dependent manner. GRE domains of Prrc2a are important for binding methylated RNA specifically, and for regulating olig2 mRNA stability, thus Prrc2a deficiency may interfere with the process of oligodendroglial specification and myelination, leading to hypomyelination. Furthermore, the interaction between Prrc2a and YTHDF2 competes for RNA bindings sites, implying that YTDHF2 may interact with Prrc2a (44). These discoveries open new avenues for investigating the fundamental mechanisms of myelination-related diseases and developing new therapeutics.

### RNA m^6^A methylation in neurological disease

#### Altered m^6^A-tagged transcript profiles in the hippocampus and cerebral cortex after traumatic brain injury

To gain insights into potential therapeutic strategies for traumatic brain injury (TBI), further investigations into its pathophysiological cascade and possible neuropathological mechanisms are needed. There is widespread public concern regarding TBI, particularly as it leads to increased mortality among young people and increases susceptibility to prolonged dysfunction, which also causes socioeconomic burdens (45, 46). TBI is grouped into primary and secondary injuries; the former manifests as cortical contusions and vascular injuries from direct hits, the latter as cerebral edema caused by a pathophysiological cascade, which may further exacerbate the primary injury and consequently aggravate neurological defects (47). m^6^A-modified transcripts have been implicated in the physiological and pathological consequences of TBI. Both expression of METTL3 and level of m^6^A in total RNA were significantly decreased in mouse hippocampus following traumatic brain injury (48). METTL3 deficiency in mouse hippocampus impairs spatial learning and memory (49). Additionally, an injury to the brain caused by TBI may result in disturbances in energy homeostasis and mitochondrial dysfunction, which may cause neurodegeneration and altered synaptic plasticity, resulting in cognitive dysfunction (50). Conjoint GO and pathway analyses of differentially methylated mRNA have shown that peaks in m^6^A-modified transcripts related to metabolic processes are significantly altered, implying that m^6^A modification is involved in altered metabolic hippocampal activity and participates in neuronal cell damage following TBI (50). Further, altered m^6^A methylation levels of some genes (e.g., cd14, Dll4, sox7) are associated with TBI pathophysiological processes (51). Lack of FTO also aggravates TBI-induced brain injuries, spatial memory problems, and neurological deficits (51).

#### Altered m^6^A-tagged transcript profiles in brain ischemia

A few studies have examined neuroinflammation and free radical production in brain ischemia, which lead to neurodegenerative disorders, destruction of blood-brain barrier integrity and even mortality (52). Neurons are more vulnerable than other cell types to hypoxic-ischemia damage, because of their lower energy compliments induced by glucose and oxygen brain reductions from relative or absolute hypoperfusion (53). Transient forebrain ischemia significantly reduces FTO expression 7–10 days after ischemia, which is associated with impaired hippocampal function (36). Further, FTO may play crucial roles in m^6^A methylation of mRNAs post-stroke, and differential m^6^A methylated RNA after focal ischemia were related with immune regulation, inflammatory signaling, and cell apoptosis (54). m^6^A methylation can be interfered with by SNPs altering the RNA sequences of target sites or key flanking nucleotides, it can affect the progression of disease consequently (55). The genome-wide association between m^6^A-SNPs and ischemic stroke by excavating data from a large-scale GWAS, rs2013162 and rs2273235 may exert its effects by altering the methylation level of m^6^A and regulating the expression of downstream gene IRF6 and NDST1 respectively, which were potential cause gene of ischemic stroke (56). Conjoint analysis of ischemic stroke genome-wide association study and mA-SNP list from the m^6^AVar database, dozens of m^6^A-SNPs were identified as functional polymorphisms and novel genetic biomarkers for ischemic stroke pathogenesis (57). YTHDC1 increases Akt phosphorylation by destabilizing PTEN mRNA and promoting degradation to assist neuronal survival, particularly after cerebral ischemia (43). These studies cumulatively show that m^6^A plays an important role in the maintenance of neurological function and that the regulation function of m^6^A may provide a potential therapeutic target for ischemic stroke.

#### Altered m^6^A-tagged transcript profiles in the brain after ischemia-reperfusion injury

Recent studies suggest that the mechanism of ischemia-reperfusion (I/R) damage involves intracellular calcium overload and reactive oxygen species (ROS) accumulation, which impair the integrity and function of mitochondria and, consequently, severely injured ischemic tissues (58). Hypothermia, the only treatment identified to ameliorate cerebral I/R injury, exerts its protective effect *via* activation of PTEN in a m^6^A-dependent manner. Additionally, through N6-methyladenosine modification of PTEN mRNA, hypothermia activates PI3K/Akt signaling, downregulates pyroptosis, and consequently plays a neuroprotective role (59). Another study showed a significant increase in m^6^A expression following OGD/R and MCAO (60). ALKBH5 and FTO selectively demethylate the anti-apoptotic protein Bcl2 transcripts, preventing degradation of Bcl2 transcripts and enhancing Bcl2 expression, and providing neuroprotective effects against I/R neuronal apoptosis (60). By targeting YTHDF1 with microRNA-421-3p, p65 mRNA is destabilized and its translation inhibited, thus preventing inflammatory responses in cerebral I/R (61).

#### Altered m^6^A-tagged transcript profiles in septic brain injury

Sepsis is caused by pathogenic infection that leads to a cascade of multi-organ inflammation and systemic immune dysfunction; progression can lead to development of severe sepsis and shock (62–64). Sepsis-related encephalopathy (SAE) can induce neuroinflammation and long-term cognitive dysfunction; its underlying mechanisms include blood-brain barrier damage and promotion of glial cell activation (65). The release of large numbers of inflammatory factors leads to inflammatory responses by the nervous system, oxidative stress and apoptosis (66). The SAE pathological process is primarily an early immune activation and late immune suppression, and is closely related to abnormal immune responses caused by dysfunction of neutrophils, macrophages/monocytes, dendritic cells and T lymphocytes (67). Mice with severe sepsis show a state of advanced immunosuppression, and immune cells (e.g., T cells, B cells) and inflammatory factors (e.g., IL-8, IL-6) are neurotoxic to the central nervous system (67), while T cell homeostasis is controlled by methylation of the m^6^A mRNA by targeting the pathways IL-7/STAT5/SOCS (68). JAK2/STAT3 expression is decreased in YTHDF1 knockout macrophages and increased IL-17 production, which is associated with the migration of inflammatory cells into the brain during sepsis. In addition, the methylation level of JAK2/STAT3 in cells reduces penetration of macrophages through the blood-brain barrier, improving neuroinflammation and reducing septic brain injury. Consequently, in YTHDF1 knockdown mouse brain tissue, the proportion of normal neurons (i.e., those that do not differ from cells exposed to severe sepsis) is significantly increase compared with mice with hyperemia (69). METTL3 inhibits the inflammation and pyroptosis through decreasing the mRNA stability of NLRP3 in LPS-treated 1321N1 cells, which exhibit a protective effect on SAE (70).

#### Altered m^6^A-tagged transcript profiles in Alzheimer's disease

Alzheimer's disease (AD) is a chronic neurodegenerative disease characterized by the accumulation of insoluble neurotoxic aggregates, primarily abnormal extraneuronal β-amyloid deposition and excess intraneuronal tau protein (71). Phosphorylation forms neurofibrillary tangles, leading to synaptic dysfunction and cognitive impairment; patient quality of life is seriously impaired before these effects lead to death (72). Though beneficial results have been achieved in rat models, the role of m^6^A methylation cannot be ignored in the pathogenic factors of AD, as it is implicated in a variety of pathological processes (73). m^6^A modification exhibits temporal and spatial specificity during brain development and is closely related to aging and neurodegenerative diseases (74). METTL3 expression is increased and FTO expression decreased in the hippocampus of AD model mice, suggesting that RNA m^6^A methylation modification promotes AD progression (73). The methylases METTL3 and RBM15B are differentially expressed in the hippocampus of patients with AD, while in the insoluble fraction, the expression level of METTL3 is positively correlated with aberrantly phosphorylated tau (75). The increasing level of m^6^A in the hippocampus and cortex from AD patients, while the m6A-related regulator proteins, like METTL3, METTL14 and FTO, significantly decreased. Besides, neurodegeneration, spine loss, and gliosis are caused by METTL3 knockdown in the hippocampus, that shMettl3 induced cell cycle abnormalities and neurodegenerative changes, and overexpression of it may protect against the Aβ-induced synaptic toxicity (76). Familial AD (5XFAD) mice have elevated METTL3 expression levels, and GO analysis shows that differentially methylated genes, including synaptic transmission and ion transport regulation, are closely related to the physiological and pathological mechanisms of AD, while m^6^A controls the protein levels of key genes, its mRNA level shows no significant change (77).

#### Altered m^6^A-tagged transcript profiles in amyotrophic lateral sclerosis

Amyotrophic lateral sclerosis (ALS) is a mortal, progressive neurodegenerative disease involving upper and lower motor neurons, which causes muscle weakness, paralysis, and ultimately death from respiratory failure (78, 79). There is currently neither cure nor treatments to slow or reverse its progression. It is influenced by environmental and genetic factors, although the exact mechanisms of these remains unknown, epigenetic alteration may be crucially involved (79–81). RBP gene mutations have been associated with neurodegenerative and psychiatric diseases, suggesting that RBP may be a cause of ALS (82, 83). Mutations of translocated in liposarcoma/beta-catenin interacts with fusion (TLS/FUS), an RNA binding protein, located in the C-terminal region (84). This contains the nuclear localization signal (NLS), which alters localization and liquid-liquid phase separation (LLPS) of TLS/FUS in affected neurons, resulting in a further shift in the TLS/FUS into cellular aggregates involved in ALS pathogenesis (85). TLS/FUS binds intensely to m6A-modified RNA fragments, suggesting that TLS/FUS may serve as m6A readers (86). Additionally, m6A-modified RNA fragments inhibit aggregation of TLS/FUS in cytoplasmic and affect localization of TLS/FUS-interacting proteins, and thus enhancing cell viability (86). Better understanding the regulation of m6A-modified RNA fragments in TLS/FUS aggregation may contribute to development of novel ALS therapeutics.

#### Altered m^6^A-tagged transcript profiles in Parkinson's disease

A relatively common neurodegenerative disease with well-defined symptoms, Parkinson's disease (PD) is characterized by bradykinesia, muscular rigidity, resting tremor and postural instability (87). Patiens with PD have progressive loss of dopaminergic neurons in the substantia nigra pars compacta and accumulation of misfolded α-synuclein protein, which mediated neurotoxicity (88, 89). Behavioral response control and neuronal activity are also impaired by the inactivation of FTO gene (90). Arsenite exposure reduced dopamine content *via* impaired dopaminergic neurotransmission and learning and memory ability (91). Additionally, increasing m^6^A level induced by arsenite exposure in male adult mice, FTO may alleviate dopaminergic neurotransmission by regulating the expression of the key protein in dopaminergic neurotransmission like TH, DAT, COMT, and DRDs (91). Integrative analysis was used to screen for candidate m^6^A PD risk loci, and identified five m^6^A-SNPs (rs75072999 of GAK, rs1378602, rs4924839 and rs8071834 of ALKBH5, and rs1033500 of C6orf10) may be associated with PD risk (92), while GAK contributes to the deposition of neurotoxic a-synuclein aggregates in PD (93). Decreased global m6A modified levels were identified in 6-OHDA-induced PC12 cells and the striatum of a rat PD model. Furthermore, overexpression of m6A demethylase FTO significantly decreases the m6A methylation in striatum and induces the expression of NMDAR1, an ionotropic glutamate receptor; its overactivation can cause neuronal injury or apoptotic cell death, resulting in amplified Ca^2+^ influx and elevated oxidative stress. These in turn ultimately lead to increased apoptosis in dopaminergic neurons. These investigators speculated that FTO could reduce the m^6^A content and enhance the mRNA stability and expression of NDMAR1 (94). As a result of excessive manganese exposure, dopaminergic neurons are damaged and DA levels are reduced in the striatum, resulting in PD; it also causes decreased FTO mRNA and protein expressions. Axon guidance molecule ephrin-B2 expression can be regulated by FTO-mediated m^6^A modification *via* YTHDF2, causing motor dysfunction in a manganese-induced PD rat model (95).

#### Altered m^6^A-tagged transcript profiles in other brain injuries

The degree of m^6^A methylation modification differs among brain injuries resulting from different diseases, but plays a crucial role in each of them. The regulatory role of m^6^A methylation modification has been confirmed in axon regeneration of the dorsal root ganglia and peripheral nerves; in addition multiple m^6^A modified genes that are differentially expressed after zebrafish spinal cord injury are all key nerve regeneration genes (28, 96).

## Conclusion and future directions

m^6^A methylation modification is the most common epigenetic post-transcriptional modification in eukaryotes. It regulates gene expression *via* biological processes like RNA splicing, nuclear export, stability and degradation, and is involved in neurodevelopment and various brain functions. Further work is needed to fully elucidate the role of m^6^A in brain development and disease. The mechanism of action of the m^6^A RNA binding protein, and how the mRNA context affects neural development and neurological disease are remain unclear. Thus, much research remains to be accomplished.

Compared with other organs, m^6^A modification is very abundant in the brain and differentially expressed in multiple brain diseases, indicating that m^6^A methylation modification is closely related to its pathophysiological mechanism. The emergence of diverse, valid sequencing technologies has broadened our understanding of the specific impact of m^6^A on brain development and disease. In brain injury, m^6^A is involved in the regulation of multiple signaling pathways, in which it can intervene.

Therefore, studying the role of m^6^A methylation in regulating the pathogenesis of brain injury and regulating m^6^A-related genes may lead to strategies for treating various brain injuries. It has great potential for application in the clinical practice of brain injury prevention and treatment. Determining the impacts of m^6^A in the brain should be a strong priority in neurobiology research.

## Author contributions

JD wrote and edited the article. AC searched for data. XC and XZ reviewed and edited the article. All authors contributed to the article and approved the submitted version.

## Funding

This research was supported by National Natural Science Foundation of China (82001166 and 82171186) and the Joint Funds for the Innovation of Science and Technology (2019Y9028 and 2019Y9023).

## Conflict of interest

The authors declare that the research was conducted in the absence of any commercial or financial relationships that could be construed as a potential conflict of interest.

## Publisher's note

All claims expressed in this article are solely those of the authors and do not necessarily represent those of their affiliated organizations, or those of the publisher, the editors and the reviewers. Any product that may be evaluated in this article, or claim that may be made by its manufacturer, is not guaranteed or endorsed by the publisher.
