# Supplementary material for: m6A RNA methylation in brain injury and neurodegenerative disease
Source: Front Neurol. 2022 Sep 8;13:995747. doi: 10.3389/fneur.2022.995747 (PMC9493472; doi:10.3389/fneur.2022.995747)
Supplement: Supplementary file 1 [file Table_1.pdf]

Nervous bioprocess/disease	Methyltransferase/ demethylases	Expression	Target RNAs	Mechanism(Refs)
Cerebellar Development	Mettl3	Downregulated	*Dapk1*,*Fadd*, *Ngfr*	Controlling mRNA stability of genes related to cerebellar development and apoptosis(1).
	Alkbh5	Downregulated	*Ddx11*,*Ccnb1*,*Cbx1*	Affecting RNA nuclear export(2).
Cortical Neurogenesis	Mettl14	Downregulated	*CBP*, *p300*	destabilizing transcripts encoding histone modifiers(3).
	FTO	Downregulated	*Pdgfra*,*Socs5-Stat3*,*BDNF*	Decreasing the expression of BDNF protiein(4).
Proliferation and Differentiation of Neural Stem Cells	FTO	Downregulated	*Pdgfra*,*Socs5*,*Stat3*	regulating aNSCs through modulating Pdgfra/Socs5-Stat3 pathway.(5).
	YTHDF2	Downregulated	*Nrp2*, *Nrxn3*	delayed mRNA clearance, contribute to the defects in neurogenesis(6).
Oligodendroglial Specification and Myelination	Prrc2a		Olig2	stabilizing Olig2 mRNA through binding to a consensus GGACU motif in the Olig2 CDS(7).
Ischemic Stroke	FTO	Downregulated	--	Reducing Fto immunoreactive structures in the hippocampus may be associated with impairments in Fto-related hippocampal function(8).
	YTHDC1	Upregulated	*Pten*	Regulating of *PTEN* mRNA stability(9).
Ischemia-Reperfusion Injury	FTO	Downregulated	*Bcl*$_2$	preventing Bcl2 transcript degradation and enhancing Bcl2 protein expression(10).
Traumatic Brain Injury	FTO/Mettl14	Downregulated		Altering transcripts related to metabolic processes(11) .
Alzheimer's Disease	FTO	Downregulated	Aβ/Synaptophysin	Enhancing Aβ protein expression and reducing synaptophysin protein expression(12).

	Mettl3	Downregulated	*tau*	level of METTL3 positively correlated with that of Tau in the insoluble fraction of these hippocampal lysates(13).
Septic Encephalopathy	YTHDF1	Upregulated	*JAK/STAT*	reducing the entry of macrophages into the brain to cause endothelial damage(14).
Spinal Cord Injury	Mettl14	Upregualted	EEF1A2	METTL14 inhibited neuronal apoptosis in the spinal cord through the mediation of EEF1A2 m6A methylation(15).
Depression	FTO	downregulated	Adrb2	ADRB2 stimulation improve the depression-like behaviors and spine loss induced by hippocampal Fto deficiency(16).

Table1. Methyltransferases/demethylases and corresponding target RNAs in brain disease and nervous bioprocess.

1. Wang CX, Cui GS, Liu X, Xu K, Wang M, Zhang XX, et al. Mettl3-Mediated M6a Modification Is Required for Cerebellar Development. *PLoS Biol* (2018) 16(6):e2004880. Epub 2018/06/08. doi: 10.1371/journal.pbio.2004880.
2. Ma C, Chang M, Lv H, Zhang ZW, Zhang W, He X, et al. Rna M(6)a Methylation Participates in Regulation of Postnatal Development of the Mouse Cerebellum. *Genome Biol* (2018) 19(1):68. Epub 2018/06/02. doi: 10.1186/s13059-018-1435-z.
3. Wang Y, Li Y, Yue M, Wang J, Kumar S, Wechsler-Reya RJ, et al. N(6)-Methyladenosine Rna Modification Regulates Embryonic Neural Stem Cell Self-Renewal through Histone Modifications. *Nat Neurosci* (2018) 21(2):195-206. Epub 2018/01/18. doi: 10.1038/s41593-017-0057-1.
4. Li L, Zang L, Zhang F, Chen J, Shen H, Shu L, et al. Fat Mass and Obesity-Associated (Fto) Protein Regulates Adult Neurogenesis. *Hum Mol Genet* (2017) 26(13):2398-411. Epub 2017/04/12. doi: 10.1093/hmg/ddx128.
5. Cao Y, Zhuang Y, Chen J, Xu W, Shou Y, Huang X, et al. Dynamic Effects of Fto in Regulating the Proliferation and Differentiation of Adult Neural Stem Cells of Mice. *Hum Mol Genet* (2020) 29(5):727-35. Epub 2019/11/22. doi: 10.1093/hmg/ddz274.
6. Li M, Zhao X, Wang W, Shi H, Pan Q, Lu Z, et al. Ythdf2-Mediated M(6)a Mrna Clearance Modulates Neural Development in Mice. *Genome Biol* (2018) 19(1):69. Epub 2018/06/02. doi: 10.1186/s13059-018-1436-y.
7. Wu R, Li A, Sun B, Sun JG, Zhang J, Zhang T, et al. A Novel M(6)a Reader Prrc2a Controls Oligodendroglial Specification and Myelination. *Cell Res* (2019) 29(1):23-41. Epub 2018/12/06. doi: 10.1038/s41422-018-0113-8.
8. Kim W, Kang MS, Kim TH, Yoo DY, Park JH, Jung HY, et al. Ischemia-Related Changes of Fat-Mass and Obesity-Associated Protein Expression in the Gerbil Hippocampus. *Metab Brain Dis* (2020) 35(2):335-42. Epub 2019/12/02. doi: 10.1007/s11011-019-00513-1.
9. Zhang Z, Wang Q, Zhao X, Shao L, Liu G, Zheng X, et al. Ythdc1 Mitigates Ischemic Stroke by Promoting Akt Phosphorylation through Destabilizing Pten Mrna. *Cell Death Dis* (2020) 11(11):977. Epub 2020/11/15. doi: 10.1038/s41419-020-03186-2.

10. Xu K, Mo Y, Li D, Yu Q, Wang L, Lin F, et al. N(6)-Methyladenosine Demethylases Alkbh5/Fto Regulate Cerebral Ischemia-Reperfusion Injury. *Ther Adv Chronic Dis* (2020) 11:2040622320916024. Epub 2020/05/20. doi: 10.1177/2040622320916024.
11. Yu J, Zhang Y, Ma H, Zeng R, Liu R, Wang P, et al. Epitranscriptomic Profiling of N6-Methyladenosine-Related Rna Methylation in Rat Cerebral Cortex Following Traumatic Brain Injury. *Mol Brain* (2020) 13(1):11. Epub 2020/01/30. doi: 10.1186/s13041-020-0554-0.
12. Han M, Liu Z, Xu Y, Liu X, Wang D, Li F, et al. Abnormality of M6a Mrna Methylation Is Involved in Alzheimer's Disease. *Frontiers in neuroscience* (2020) 14:98. Epub 2020/03/19. doi: 10.3389/fnins.2020.00098.
13. Huang H, Camats-Perna J, Medeiros R, Anggono V, Widagdo J. Altered Expression of the M6a Methyltransferase Mettl3 in Alzheimer's Disease. *eNeuro* (2020) 7(5). Epub 2020/08/28. doi: 10.1523/eneuro.0125-20.2020.
14. Xing Y, Cheng D, Shi C, Shen Z. The Protective Role of Ythdf1-Knock Down Macrophages on the Immune Paralysis of Severe Sepsis Rats with Ecmo. *Microvascular research* (2021) 137:104178. Epub 2021/05/21. doi: 10.1016/j.mvr.2021.104178.
15. Gao G, Duan Y, Chang F, Zhang T, Huang X, Yu C. Mettl14 Promotes Apoptosis of Spinal Cord Neurons by Inducing Eef1a2 M6a Methylation in Spinal Cord Injury. *Cell death discovery* (2022) 8(1):15. Epub 2022/01/12. doi: 10.1038/s41420-021-00808-2.
16. Liu S, Xiu J, Zhu C, Meng K, Li C, Han R, et al. Fat Mass and Obesity-Associated Protein Regulates Rna Methylation Associated with Depression-Like Behavior in Mice. *Nat Commun* (2021) 12(1):6937. Epub 2021/11/28. doi: 10.1038/s41467-021-27044-7.
